# Supplementary material for: FastqPuri: high-performance preprocessing of RNA-seq data
Source: BMC Bioinformatics. 2019 May 3;20:226. doi: 10.1186/s12859-019-2799-0 (PMC6500068; doi:10.1186/s12859-019-2799-0)
Supplement: Supplementary file 2 — Archive of FastqPuri. Archive containing all files needed to install and run FastqPuri v1.0.6. Date stamp March 22, 2019. (GZ 47,819 kb) [file 12859_2019_2799_MOESM2_ESM.gz › FastqPuri-1.0.6/html/struct__fq__read.html]

FastqPuri: \_fq\_read Struct Reference


FastqPuri

Public Attributes |
List of all members

\_fq\_read Struct Reference

stores a fastq entry
More...

`#include <fq_read.h>`

|  |  |
| --- | --- |
| Public Attributes | |
| char | line1 [READ\_MAXLEN] |
|  | |
| char | line2 [READ\_MAXLEN] |
|  | |
| char | line3 [READ\_MAXLEN] |
|  | |
| char | line4 [READ\_MAXLEN] |
|  | |
| int | L |
|  | |
| int | start |
|  | |
| int | Lhalf |
|  | |
| char | extended [READ\_MAXLEN] |
|  | |
| unsigned char | pack [(READ\_MAXLEN+1)/2] |
|  | |
| unsigned char | packsh [(READ\_MAXLEN+1)/2] |
|  | |
| int | L\_ad |
|  | |
| int | L\_ext |
|  | |
| int | L\_pack |
|  | |
| int | L\_packsh |
|  | |

## Detailed Description

stores a fastq entry

## Member Data Documentation

## ◆ extended

char \_fq\_read::extended[READ\_MAXLEN]

extended sequence, adapter added to 5' end

## ◆ L

int \_fq\_read::L

read length

## ◆ L\_ad

int \_fq\_read::L\_ad

length of adapter sequence

## ◆ L\_ext

int \_fq\_read::L\_ext

length of extended sequence

## ◆ L\_pack

int \_fq\_read::L\_pack

length of packed sequence

## ◆ L\_packsh

int \_fq\_read::L\_packsh

length of packed sequence (shifted)

## ◆ Lhalf

int \_fq\_read::Lhalf

half of read length

## ◆ line1

char \_fq\_read::line1[READ\_MAXLEN]

Line 1 in fastq entry

## ◆ line2

char \_fq\_read::line2[READ\_MAXLEN]

Line 2 in fastq entry

## ◆ line3

char \_fq\_read::line3[READ\_MAXLEN]

Line 3 in fastq entry

## ◆ line4

char \_fq\_read::line4[READ\_MAXLEN]

Line 4 in fastq entry

## ◆ pack

unsigned char \_fq\_read::pack[(READ\_MAXLEN+1)/2]

pack sequence

## ◆ packsh

unsigned char \_fq\_read::packsh[(READ\_MAXLEN+1)/2]

pack sequence with shift

## ◆ start

int \_fq\_read::start

nucleotide position start. Can only be different from zero if the read has been filtered with this tool.

---

The documentation for this struct was generated from the following file:

- include/fq\_read.h


---

Generated on Mon Mar 19 2018 23:42:01 for FastqPuri by  

 1.8.14
